# Biosensing and Actuation—Platforms Coupling Body Input-Output Modalities for Affective Technologies

**DOI:** 10.3390/s20215968

**Published:** 2020-10-22

**Authors:** Miquel Alfaras, William Primett, Muhammad Umair, Charles Windlin, Pavel Karpashevich, Niaz Chalabianloo, Dionne Bowie, Corina Sas, Pedro Sanches, Kristina Höök, Cem Ersoy, Hugo Gamboa

**Affiliations:** 1 PLUX Wireless Biosignals, Avenida 5 de Outubro 70, 1050-059 Lisboa, Portugal; wprimett@plux.info; 2 Departament d’Enginyeria i Ciència dels Computadors, RobInLab, Universitat Jaume I, Avinguda de Vicent Sos Baynat, s/n, 12071 Castelló, Spain; 3 Departamento de Física, LIBPhys FCT—UNL Universidade NOVA de Lisboa, Largo da Torre, 2825-149 Caparica, Portugal; hgamboa@fct.unl.pt; 4 Computing and Communications Department, InfoLab21, Lancaster University, Bailrigg, Lancaster LA1 4WA, UK; m.umair7@lancaster.ac.uk (M.U.); dionne.bowie@nhs.net (D.B.); c.sas@lancaster.ac.uk (C.S.); 5 Division of Media Technology and Interaction Design, School of Electrical Engineering and Computer Science, KTH Royal Institute of Technology, Brinellvägen 8, 114 28 Stockholm, Sweden; windlin@kth.se (C.W.); pavelka@kth.se (P.K.); sanches@kth.se (P.S.); khook@kth.se (K.H.); 6 Computer Engineering Department, Boğaziçi University, Rumeli Hisarı, 34470 Sarıyer/Istanbul, Turkey; niaz.chalabianloo@boun.edu.tr (N.C.); ersoy@boun.edu.tr (C.E.); 7 Research and Innovation Centre, Leeds Teaching Hospitals NHS Trust, Beckett St, Leeds LS9 7TF, UK

**Keywords:** human-computer interaction, affective technologies, interaction design, biosensing, actuation, somaesthetics, design toolkits

## Abstract

Research in the use of ubiquitous technologies, tracking systems and wearables within mental health domains is on the rise. In recent years, affective technologies have gained traction and garnered the interest of interdisciplinary fields as the research on such technologies matured. However, while the role of movement and bodily experience to affective experience is well-established, how to best address movement and engagement beyond measuring cues and signals in technology-driven interactions has been unclear. In a joint industry-academia effort, we aim to remodel how affective technologies can help address body and emotional self-awareness. We present an overview of biosignals that have become standard in low-cost physiological monitoring and show how these can be matched with methods and engagements used by interaction designers skilled in designing for *bodily engagement* and *aesthetic* experiences. Taking both strands of work together offers unprecedented design opportunities that inspire further research. Through *first-person soma design*, an approach that draws upon the designer’s felt experience and puts the sentient body at the forefront, we outline a comprehensive work for the creation of novel interactions in the form of couplings that combine biosensing and body feedback modalities of relevance to affective health. These couplings lie within the creation of design toolkits that have the potential to render rich embodied interactions to the designer/user. As a result we introduce the concept of “*orchestration*”. By orchestration, we refer to the design of the overall interaction: coupling sensors to actuation of relevance to the affective experience; initiating and closing the interaction; habituating; helping improve on the users’ body awareness and engagement with emotional experiences; soothing, calming, or energising, depending on the affective health condition and the intentions of the designer. Through the creation of a range of prototypes and couplings we elicited requirements on broader orchestration mechanisms. First-person soma design lets researchers look afresh at biosignals that, when experienced through the body, are called to reshape affective technologies with novel ways to interpret biodata, feel it, understand it and reflect upon our bodies.

## 1. Introduction

The rise of tracking technologies has started to foster international collaborations that tackle the design of technologies for emotional awareness and regulation to support wellbeing and affective health. In fact, mental health research is trying to catch up with the affordances that ubiquitous technologies, wearable devices, and tracking systems offer in general, albeit not without challenges [1,2,3]. These can be addressed through interdisciplinary research bridging the gap between the fields of Human-Computer Interaction (HCI), Biosensing research, and Clinical psychology [4,5,6]. Projects such as AffecTech [6,7], have explored the development of digital platforms that position bodily affective awareness and engagements centrally, drawing on *somaesthetic design* [8]. Somaesthetic, or soma design for short, is grounded in somatic experiences, letting designers examine and improve on connections between sensation, feeling, emotion, subjective understanding and values. The soma design framework offers a coherent theoretical basis starting from the constitution and morphology of our human body and perception [8,9,10]. In particular, soma design emphasizes our ability to change and improve our aesthetic appreciation skills and perception. Other interaction design works within affecting computing exemplify ways to draw upon the body and its embodied metaphors [11,12]. This research builds on the growing HCI interest in affective technologies, whose ethical underpinnings could benefit from more consideration [13,14], addressing issues such as the pluralism of bodies, data privacy and ownership. The body has for a long time inspired emotion research across disciplines [15,16,17,18], as relevant connections exist between the body, emotion and movement, its interpretation, enacting and processing. Moreover, research studies point at the links between emotion and physical activity, for example, dance, exercise, movement, or paying attention to our body senses while immersed in nature [19,20,21]. Engagement with and through the body might therefore be a fruitful path to explore. There is room for an affective computing that does not look at the body as “an instrument or object for the mind, passively receiving sign and signals, but not actively being part of producing them”—as phrased by Höök when referring to dominant paradigms in commercial sports applications [22]. However, how to best address bodily movement and engagement beyond measuring cues and signals is unclear. Most studies in affective computing revolve around affect recognition from emotion detection and bodily data classification [23]. We take somaesthetic design—a design stance that draws upon the felt body and takes inspiration from experiencing it—and then combine it with the innovative integration of biosensors and actuators. The disruptive somaesthetic view, moves away from the idea of monitoring the body for the sake of bad habit reduction in pursuit of a healthy and long life [24]. Soma design, rather, lets us get attuned to our bodies and use sensations as a valuable resource instead of something to be improved to meet performance standards.

In this context, we present novel research on embodied interaction design couplings, that is, sensing-actuation combinations of aesthetically evocative body input-output modalities that render biodata shareable, body-centered, highly tangible or even able to be experienced collectively. The biosignals we address have become standard for physiological data tracking research, and are present in the low-cost BITalino biosensing platform [25]. Choosing them for the overview presented and further exploration is motivated by these two aspects, that is, standard and low-cost. The contribution of this paper is an approach to designing sensing-actuation orchestrations, that is, the ways in which body input-output systems and meanings are put in place, coupled, coordinated, customized, sequenced and exposed so that the underlying mechanisms can be better understood, challenged or extended—in other words, sketching, in hardware and software, tangible experiences that allow designers to design and improve overall orchestrated experiences addressing affective health. To make this design approach viable, we combine:

1. An overview description of biosignals used in ubiquitous low-cost personal sensing technologies that have gained strength in affective technology research and the possible actuation feedback elements that, when coupled, can lead to novel interactions embracing body awareness
2. The use of soma design [8], with an evaluative and explorative stance, to assess whether couplings are meaningful or evocative to address the question of how interaction design can further support interdisciplinary research of affective technologies

The individual components that take part in an interaction that integrates different body inputs and outputs must implement ways to communicate information, process and represent it, trigger events turning actions on/off and enabling interaction decisions. An orchestration of the protocols and interfaces involved could be beneficial for the design exploration or even for the introduction of use case scenarios, for example, closer to the actual psychotherapeutic practice [2,3,26]. In the discussion, we describe how these elements are shaping the future direction of our research, for example, extending interaction configuration tools with novel sensing-actuation couplings to better explore the design space of affective health technologies and their ethical underpinnings. Using technology for sensing and actuating upon our body, we can get access to bodily states from our physiology to then act in such a way that we help to alter or reassess our psychophysiological states. This construction process may be developed to extend our knowledge and expectations regarding the internal mechanics of our own body and serves as a bridge to design better informed affective health technologies. Moreover, not only does this approach aim to help having a better self-understanding but paves the way to put the body and its felt experience at the core of the design of such technologies.

The paper is organized as follows. In Section 2, we provide a brief overview of self-monitoring and affective technologies. Section 3 showcases a set of biosignals that we have had access to throughout our research and the information we can extract from their features in order to open a window to our internal psychophysiological processes. The features commonly available for each biosignal are listed to provide guidelines on what level of information is to be extracted. The actuation on the subject’s body, addressed in Section 4, can be executed through a variety of mechanisms. We list actuation mechanisms that are available for interaction design using mainly consumer electronics. In Section 5, we present the design research approach that we have adopted, describing what the first-person perspective is and introducing soma design in this context. This design process has been applied to several explorations. The outcomes of our design explorations, coupling biosensing to actuation, are discussed (see Section 6). In this section, together with Section 7, we proceed by addressing coupling concepts and discussing the orchestration process. With the idea of orchestration, we highlight the role of technology-coordinated sequences and the possibilities brought by machine learning and advanced signal processing. We end by commenting on the ethical underpinnings of affective technology and somaesthetic design.

## 2. Body-Centric Affective Technologies

With the emergence of everyday personal sensing such as the sensing embedded in our permanently reachable phones, smart watches and fitness bracelets, HCI and ubiquitous computing scholars have highlighted the value of these technologies for innovative research. *Affective Computing* refers to computing that relates to, arises from, or deliberately influences emotions [27]. Technologies that we have seen permeate the *everyday* space with quantification, exercise tracking, and physical wellbeing, have also—perhaps in line with a more traditional affective computing view—made researchers dream of extended healthcare, diagnosis and monitoring applied as well to mental wellbeing [4,28,29,30]. As exemplified by Bardram and Matic [1], mental health research is catching up. In recent years, research on mobile and wearable technologies that track behavioral, psychological, and contextual signals has gained momentum in the field, albeit not without pending design challenges [31]. Following a research path toward ubiquitous technologies deployed in mental wellbeing domains may help to bring attention to such aspects as personalization, achieving forms of rapport or engagement not seen in traditional healthcare. The promise of affective computing is vast. In our view, we could argue that just as self-awareness plays a major role in the motivation of change in rehabilitation therapy, for example, in cardiac rehabilitation [32], psychotherapy could benefit from self-monitoring technologies revealing bodily dynamics. Awareness, for instance, may contribute both to a (re)assessment of emotions and behavioral change that are solid grounds of cognitive behavioral psychotherapy [33,34].

Emotion plays an integral role in design work, and design researchers are not exempt from its ups and downs [35,36,37]. As affective computing reaches maturity, alternative methods have emerged and reshaped traditional approaches to affect. In an effort to attend to emotions, rather than primarily recognizing them, researchers investigating what is known as the affect through interaction [22] prioritize making emotion available for reflection. In such line of thought, seeking emotion aside from context would not make sense. In this “*affect-through-interaction*” view, the role that emotion has had for a long time in artistic and design endeavors is acknowledged. This is exemplified by the analysis of Boehner et al. [38], later picked up by Howell et al. [39] to defy the role of personal sensing in design, in particular the role of biosensing. That is, by no means, to say that the progress that personal sensing has witnessed under the advent of affective computing should be diminished. Rather, dialogue with artificial intelligence research and attention to more cognitivist-oriented outcomes can strengthen the affect-interaction paradigm. From our standpoint, when designing technology-mediated experiences, we see the affect as a sociocultural, embodied, and interpretative construct. Hence, embarking on the challenge of creating use cases for novel technology that touch upon emotions, we start experiencing the body first (see Section 5). The examples and reflections laid down in this paper, the description of technologies we choose to design with, our AffecTech coupling results, and those we used as inspiration, convey directions in which we believe personal sensing, its mapping to actuation, and designing with the body are successfully integrated. Under the overarching lens of first-person design that provides strong foundations, paying respect to ethics, and “resisting the urge” [35] to engage users, we rediscover (and invite others to do so) technologies that are called upon to extend possibilities within affective interaction.

### State of the Art

In the design space of affective interaction and physiological data, existing research has utilized visual and haptic technologies for affective feedback. *Affective Health* [40], for example, mapped skin conductance data measured from an electrodermal activity sensor (EDA) into a colorful spiral on a mobile phone screen. After using the mobile app for a month, users interpreted the skin conductance data as a tool to manage stress levels, track emotions, monitor personality, and even to change their behaviors. Khut [41] has been a pioneer in the area of designing heart rate based visual and sonic artworks for relaxation, both through a mobile app and large scale projections. HCI researchers have started to utilize alternative materials such as thermochromic ones to visually represent biosensing data. Howell et al. designed Ripple [42], a thermochromic-based shirt that changes colors responding to skin conductance. By using the garment over a two-day period, wearers were able to reflect on their emotions but they rarely questioned if the display was actually representing their feelings. In Reference [43], Umair et al. mapped skin conductance to haptic changes in addition to using visual thermochromic materials, that is, vibrations, heating, and squeezing effects. The feedback about the body properties measured is worn, felt or placed in contact with the body. The findings of these studies highlight that the material-driven qualities of such visual and haptic body interactions shape people’s interpretation of how they identify, attribute, and regulate emotions in everyday life. Haptics have also been used with biosensors to regulate affect, which requires users to adapt their ongoing feelings. *EmotionCheck* [44] and *Doppel* [45] use vibrations simulating a slower heart rate sensation for the users and helped them decrease their anxiety. Recently, Miri et al. [46] used a vibration-based personalized slow-paced breathing pacer on the belly which delivered vibrations in a biphasic pattern for inhalation and exhalation and helped users in reducing anxiety during a stressor. With a research approach that explicitly sets out to design with the body—not as an object to be measured but “understanding the body as a site of creative thinking and imagination” [47]—, works at the intersection of biosensing, interaction design and affective technologies offer an opportunity to study how to support the design of interactions that make us connect with our bodies [9,48,49].

## 3. Sensing the Body

Biosignals are time representations of changes in energy produced in the body. These changes correspond to energy variations of different nature, such as electrical, chemical, mechanical, and thermal (as presented in Table 1). With the turn of the 21st century and the advent of the digital era, the advances in the field of electronic components that spurred the development of computing, instrumentation, and algorithms left their impact on medical and biosignal devices. Biosensing and electrophysiology technologies were greatly improved, ready for the study of body functions and health monitoring in the context of clinical research. As technologies grew, the miniaturization and reduction of costs contributed to the growth of biosensing monitoring technologies beyond clinical settings as well. Physiology signals and sources of tracking information are more available than ever, ranging from electromyography (EMG), electrocardiography (ECG), electroencephalography (EEG), electrodermal activity (EDA) to electrooculography (EOG) or eye movement tracking.

A direct consequence of such rapid expansion is the creation of the sports & health monitoring markets that fill up the mobile app stores and provide remarkable revenues in the ubiquitous computing paradigm that we live in. The democratization of the study of biosignals, however, comes with interesting possibilities such as a better understanding of the self and a richer, unprecedented way to interact with technologies that accompany us. This yields an opportunity to define alternative ways to live an affectively healthy life.

As the maturity of open access physiology databases [51] backs up the improvement of processing algorithms, low-cost hardware platforms help populate the open source space [52] where users embrace biosensing, share ideas and drive the future of biosignals applied in different areas. Furthermore, the biosignals that were once limited to hospitals and clinics, or in specialized research labs, addressed in classical texts of physiology, are nowadays accessible in virtually any context by means of wearable technologies. In the review of Heikenfeld et al. [53], an interesting account of the transition from lab tracking to wearables during the 20th century is offered along an in-depth overview of body sensing mechanisms not only restricted to electrophysiology. The field of affective computing has consistently found in biosignals a relevant source of information [54]. Besides, the fact that biosensing platforms have jumped off the clinic has contributed to embracing them alongside other technologies like movement tracking, traditionally linked to behavioral and psychophysiology labs.

**Figure 1:**
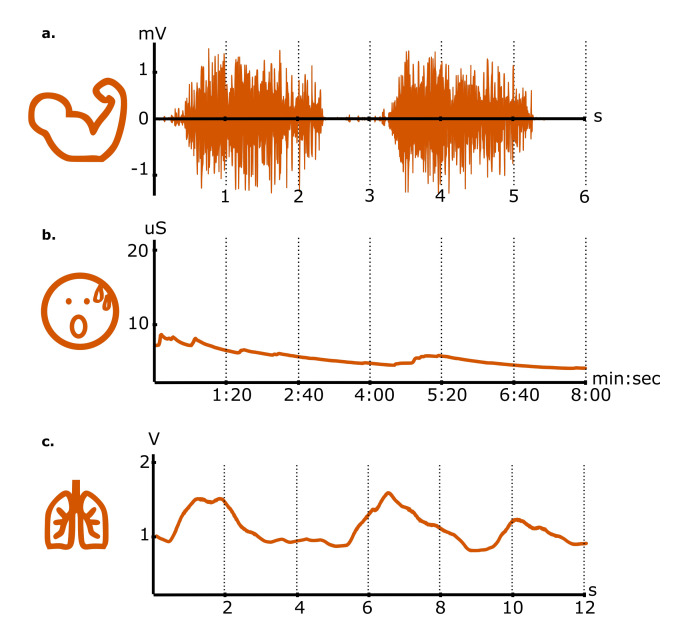
Visual representation of different biosignals: (**a**) Electromyography (EMG), (**b**) Electrodermal activity (EDA) and (**c**) Respiration signals. biosignals and icons obtained at PLUX S.A.

We present a selection of studied biosignals (see Figure 1) that can be incorporated into the creation of new technologies for affective health. We focus on a subset of biosignals present in the BITalino revolution do-it-yourself (DIY) low-cost biosensing platform [25,55,56] that backed and inspired some of our research in affective technologies. These, although not an exhaustive list, are to some extent physiological signals that have become standard for physiology tracking research—slowly crossing disciplines and making their way into affective health tracking, interaction design, and other domains of interest. Moreover, with objectives that range from out-of-the-lab psychophysiology tracking [57,58,59] to new perspectives in interaction design [43,49,60] our work has often addressed biosignals through other available biosignal research platforms beyond BITalino, such as biosignalsplux [61], Empatica E4 [62], Arduino accessories like the Grove GSR [63], or even commercial wearables such as the Samsung Gear S2 [64] among others.

In this section we present a collection of these biosignals and offer a systematic but brief description on (1) How it works, summarizing the basic physiological principles that provide the biosignals energy observables; (2) What can be extracted from the collected biosignal; (3) Where the biosignal can typically be collected in the human body; (4) When, or how often, the signal should be sampled describing the concerns on the timing of the acquisition and in particular the typical sampling frequency of each biosignal; and (5) Limitations of the biosignal acquisition and processing with the challenges of noise or signal artifacts. All of them are examples of signals that we have addressed in our research. This non-exhaustive selection offers a good starting point for researchers interested to integrate biosignals in their design of technologies for wellbeing and mental health.

### 3.1. Surface Electromyography (sEMG)

**How it works:** The recording of the electrical activity produced by skeletal muscles receives the name of electromyography (EMG). Human muscles are made up of groups of muscle units that, when stimulated electrically by a neural signal, produce a contraction. The recording of the electrical activity of the muscles (voltage along time), traditionally relying on intrusive needle electrodes (intramuscular), is easily accessible nowadays by means of surface electrodes that capture the potentials of the fibers they lay upon. The result of this measurement is a complex surface electromyography signal (sEMG) that reveals data about movement and biomechanics of the contracted muscles (see Figure 1a).

**What:** Electromyography signals inform about the contraction of specific muscles and parts of the body. The EMG signal consists in the time representation of rapid voltage oscillations. Its amplitude range is approximately 5 mV. In terms of signal analysis, the EMG allows the assessment of several aspects such as muscle contraction duration, the specific timing at which movements or contractions are activated, the presence of muscular tension or fatigue, and the extent to which different fibers (area) are contracted. The analysis is conducted through noise filtering, together with feature extraction that yields contraction onset detection, the estimation of signal envelopes, and the computation of average frequencies. This lets subjects deepen their understanding of movement strategies, very relevant for embodied art and sports performance, improve muscle coordination, or even reveal existing movement patterns that they are unaware of.

**Features:** Onset instants; Max amplitude; Instant of maximum amplitude; Activation energy; Envelope.

**Where:** Having become the standard in EMG monitoring, bipolar surface electrodes consist of three electrodes. Two of them (+/−) must be placed close to each other, on the skin that lies on top of the muscle under study, along the fibers’ direction, while the third one is placed in a bony area where no muscular activity is present. This allows the measurement of electrical potential differences with respect to a common reference, yielding a unique signal that represents the muscular activity of the area.

**When/Frequency:** Given the fast muscle-neural activation nature of EMG signals and the presence of different active muscles contributing to the same signal, muscle activity must be acquired at sampling rates no lower than 200 Hz frequencies. Working at 500 Hz is desirable, while a sampling rate of 1000 Hz guarantees the tracking of all the relevant events at a muscular level.

**Limitations:** Surface EMGs are intrinsically limited to the access to superficial muscles. This is compromised by the depth of the subcutaneous tissue at the site of the recording which depends on the weight of the subject, and cannot unequivocally discriminate between the discharges of adjacent muscles. Proper grounding (reference electrode attached to a bony inactive muscular region) is paramount to obtain reliable measurements. Motion artifacts and muscular crosstalk compromise the assessment of the muscle activity under study. In this context, interference from cardiovascular activity is not uncommon, particularly in areas such as chest and abdomen. The presence of power supplies and mains (powerline) in the vicinity poses the risk of 50 Hz–60 Hz interference.

### 3.2. Electrodermal Activity (EDA)

**How it works:** Electrodermal activity (EDA), also known as galvanic skin response (GSR), measures the electrical properties of the skin, linked to the activation of the autonomic nervous system (or more precisely the sympathetic nervous system). By applying a weak current upon two electrodes attached to the skin, it is possible to measure the variations of voltage that are present between the measuring points (see Figure 1b). When placed at specific locations on the skin, the measured electrical signals are affected by the sweat secreted by the glands that are found in the dermis.

**What:** Electrodermal activity signals inform about the activity of the sympathetic nervous system. Given its electrolyte composition, the sweat secreted by sweat glands has an impact on the electrical properties of the skin. This phenomenon, visibly monitored in voltage signals by means of electrical conductance (or impedance/resistance, conversely), facilitates the assessment of arousal effects. Arousal is the physiological response that stimuli such as emotional or cognitive stressors trigger. The measurement of electrodermal activity is usually decomposed in two major behaviors present and superposed in any skin response signal, that is, the skin conductance (tonic) level, with slowly varying dynamics, and the skin conductance (phasic) responses, that exhibit relatively faster dynamics. In terms of signal analysis, this decomposition is accompanied by the assessment of characteristics such as the rate of detected EDA events, detection of onsets, and the characteristic rise and recovery times.

**Features:** Onset instant; Skin Conductance Response (SCR) rise time; SCR 50% recovery time; Event rate; Skin Conductance Level (SCL).

**Where:** EDA measurements use two electrodes to monitor changes in electric potential between two locations on the skin. Electrodes must be placed a few centimeters apart for differences to be relevant. The nature of the measurement technique and the phenomenon itself, makes hand palm a suitable electrode location, for which either palm placement or finger phalanges, most subject to skin sweating, are optimal for the monitoring of electrodermal activity. Additionally, foot sole placement, also affected by sweating glands, is not uncommon in EDA measurements given that particular use cases or settings require access to hands for carrying out certain activities. For the alternative placements of the EDA sensors, such as forehead or wrist, the presence (or lack) of sweating glands remains a decisive factor in obtaining reliable measurements.

**When/Frequency:** Electrodermal activity is considered to be a slow physiological signal. Thus, sampling rate frequencies as low as 10 Hz allow a full representation of the skin conductance variations. Electrodermal activity peaks usually occur after few seconds from the exposure to a given stimulus (1–5 s).

**Limitations:** Electrodermal activity measurements use changes in electrical properties of the skin produced by sweating. Since sweating is not only triggered by arousal but also the human thermoregulation system, ambient heat and physical activity monitoring are aspects that limit the capabilities of EDA studies. In common practice, electrodermal sensors are usually prepared to obtain salient data from the most comprehensive userbase, providing relevant (measurable) changes regardless of the wide variety of sweating responses from subject to subject. However, it is not uncommon to find examples of subjects with either too high or too low skin conductance responses that complicate the measurements. Moreover, settings that involve an intense physical activity pose concerns on the electrode attachment and motion interference. The presence of power supplies and mains (power line) in the vicinity of the acquisition systems pose the risk of 50 Hz–60 Hz interference. With regard to feasibility, since traditional electrodermal activity studies rely on hands or feet electrode placement that compromises certain actions, attention needs to be given to the use case and activities that take place while monitoring, on a case by case basis.

### 3.3. Breathing Activity

**How it works:** Respiration (or breathing) sensors monitor the inhalation-exhalation cycles of breathing, that is, the process to facilitate the gas exchange that takes place in the lungs. In every breathing cycle, the air is moved into and out of the lungs. A breathing sensor uses either piezoelectric effects on bendable wearable bands or accessories (one of the most predominantly used technologies), respiratory inductance plethysmography on wired respiration bands around the thorax, microphonics on the nose/mouth airflow, plethysmographs (measuring air inflow) or radiofrequency, image and ultrasonic approaches. A review on breathing monitoring mechanisms is found in Reference [65]. For piezoelectric breathing sensors, thoracic or abdominal displacements (strain) produced in breathing cycles bend a contact surface that converts ressistive changes to continuous electrical signals (see Figure 1c).

**What:** A breathing signal informs about the respiration dynamics, that is, the dynamics of the process mediating gas exchange in the lungs, as well as supporting sound and speech production. The monitoring of the fundamental function of breathing brings in the assessment of breathing cycles and rates which in turn allows the study of apnoea-related problems (involving breathing interruptions), oxygen intake, metabolism of physical activity, and the effect of cognitive or emotional stressors in breathing. In terms of analysis, breathing cycles are studied using breathing rates, the maximum relative amplitude of the cycle, inhale-exhale volume estimation, inhale-exhale duration, and inspiration depth, that allow the characterization of several breathing patterns.

**Features:** Respiration rate; Inspiration duration; expiration duration; Inspiration-expiration ratio; Inspiration depth.

**Where:** A piezoelectric breathing sensor is usually located on the thoracic cavity or the belly, using a wearable elastic band. With adjustable strap and fastening mechanisms, the sensor can be placed slightly on one side where bending is most relevant, optimizing the use of the sensor range. These kinds of sensors, allow both the study of thoracic and abdominal breathing. With the development of conductive fabric, breathing sensors are making its way into the smart garment market in the form of T-shirts and underwear bands.

**When/Frequency:** Breathing is a relatively slow biosignal, with breathing rates often below 20 inhale/exhales per minute. A sampling rate frequency as low as 50 Hz is sufficient to capture the dynamics of respiration.

**Limitations:** While piezoelectric breathing sensors are prominent given the low cost and form factor advantages of wearable sensor platforms, deviations in placement have an effect in the relative range of the response signal. Movement artifacts, most relevant when physical activity is present, are a common source of problems. Respiration sensing techniques like the respiratory inductance plethysmography, compensate the highly localized piezoelectric approach with a sensor capturing the general displacement of the whole thoracic cavity, yielding a signal less prone to movement artifacts. The monitoring of breathing cycles is usually accurate, although the exploration of effects to be used as voluntary inputs in interactions, such as holding the breath, are not easily captured.

## 4. Actuation

The mechanisms to provide actuation in a form of feedback to the human take an important role in creating a complete interaction from sensing body properties to making the subject aware of them. Our research aims at linking biosensing to body actuation. Actuation is generally provided by mechanical elements that move and respond to input signals in order to either control or inform about a system. We stretch this definition to include feedback mechanisms such as screen-based visuals, although no mobile mechanical element is necessarily implied. In this section, we focus on actuation mechanisms that can be easily controlled and coupled to our body. We take a similar approach to the structure used to describe the biosignals, on explaining: how, what, where, when, and the actuator limitations and usage precautions for a selected list of actuators. The range of actuation mechanisms presented draws upon our research on affective technologies and interaction design, as well as inspirational works present interaction design research, but it should be seen as a non-exhaustive list of possibilities.

### 4.1. Screen-Based Visual Biofeedback

**How it works:** Screen-based visual biofeedback is the representation of body signals that inform about body changes happening along time. Its goal is to provide to the researcher a means to assess the dynamics of the aforementioned changes, helping to gain understanding and tracking the inner state of a given subject. Examples of this could be ECG feedback, respiration feedback, or movement tracking, usually employed in health metrics or sports performance research. Screen-based biosensing systems for feedback are standard practice in clinical settings and hospitals. Biofeedback use has for instance been adopted in psychotherapy, as research suggests that the technique provides a mechanism to self-regulate the emotions.

**What:** Screen-based visual biofeedback uses a 2D graphical interface and benefits from light, colors, strokes, and visual styles to represent a changing signal that evolves with time. Signal peaks and troughs appear in an axis showing the measurement magnitude in a given range, so that rapid and slow dynamics can be seen as the representation moves along the time axis when updated.

**Where:** Screen-based visual feedback takes place in a display, either a computer screen or a sensing platform display.

**When:** It is important that the represented signals are updated in real-time. Doing otherwise, although possible using delays or technology limitations, would compromise the ability of the actuation to convey the tracking meaning attributed to the practice of biofeedback. When sensing requirements pose concerns on the technical ability to render a smooth representation through time, approaches such as averaging or undersampled representations are used.

**Limitations:** Screen-based visual biofeedback connects easily with the mathematical properties that underlie the signals under study. However, signal processing procedures such as filtering, scaling or normalization are crucial in achieving a smooth and flowing representation. These come, of course, tightly dependent on the available computing capabilities. There are situations in which feedback users report finding difficulties or experiencing anxiety when engaging in the assessment of body rhythms. Moreover, visual information tends to remarkably capture the attention of the user, thus needing special care when used as an element of broader interaction (movement, performance, exercise) that could render a poorer experience quality or present a deviation from the aimed activity.

### 4.2. Sound Feedback

**How it works:** Sound feedback, when applied to biosignals, is the audio representation of body signals that uses sound properties to inform about body changes happening along time. Its goal is to exploit our sophisticated trained sense of hearing to convey meanings linked to body signal features, leading to the understanding and tracking of a given subject’s biosignal dynamics.

**What:** Sound feedback uses the properties of sound, that is, volume, pitch or frequency (note), rhythm, harmony, timbre, and transients (attack, sustain, etc.) among others, to represent a signal (or its features) that changes over time. Its generation, often using speakers or headphones, is linked to properties of the signal. Alternative approaches draw upon several transducing paradigms, that is, different ways to convert electrical signals into sound (electromechanical as in the case of speakers, piezoelectric or others), often more limited such as buzzers or beepers made of basic vibrating elements that produce sound.

**Where:** Sound can be generated in speakers, devices that work converting electrical pulses to sound (air pressure) waves, allowing users to listen to the feedback without the need for additional equipment. Headphones, working by the same principle, can be used for the same purpose but only providing feedback to the person wearing them.

**When:** The human hearing range typically comprises frequencies between 20 Hz and 20,000 Hz. The oscillating frequency of the sound wave that is created is what gives it a particular tone (what we call a note). The different times at which sound waves are generated is what creates the meaning of rhythm and articulation.

**Limitations:** Audio generation and processing techniques are complex. Whilst high-level hardware and software tools can be exploited to make a complete system more accessible, there certainly remains a relevant learning-curve. The scenario in which audio feedback is deployed conditions a lot the effect achieved, given the fact that materials surrounding the sound generating system at use impose effects like reverberation, echoes, or absorption. Exposure to sound feedback for a prolonged period of time has some drawbacks. Sound volume can potentially harm our auditory system. Sound feedback that lack textural richness (e.g., a single sine-wave) has the risk of becoming unengaging for the user or potentially cause irritation.

### 4.3. Vibrotactile Actuation

**How it works:** Vibrotactile actuation uses motors to stimulate communication utilizing touch, and more precisely tactile vibrations. When linked to biosensors, it can use the properties of the so-called vibrations to convey features of the biosignal being tracked.

**What:** Vibrotactile actuation is a technology communication mechanism that uses touch vibrations to exploit the touching sense of humans. It is built upon motors, which can mostly be categorized under two types:

- Eccentric rotating mass vibration motor (ERM), with a small unbalanced mass on a DC motor that creates a centripetal force translated to vibrations when rotating.
- Linear resonant actuator (LRA), containing a small internal magnetic mass attached to a spring, which creates a displacement force in a single axis when driven by an AC signal, usually operating around a specific narrow frequency bandwidth that increases efficiency.

These motors usually take the form of small (few millimeters) enclosures with simple positive and negative (+/−) terminals to be driven, lowering the power and supporting (weight) requirements. The typical power supply needed for this kind of micromotors is of the order of 1–5 V.

**Where:** With weights below 1g, the small form factor of these motors makes them suitable for body explorations, often relying on patches, elastic bands, or holders. Typical uses include also vibrotactile-equipped wristbands or smartwatches. Besides the traditional game/remote controllers including vibrotactile feedback and actuating on the hands, the currently ubiquitous role of mobile phones has spread the use of vibration feedback and patterns for notification, alarms and other communication examples anywhere a phone can be placed or hold.

**When:** Small vibrotactile motors feature fast startup and breaking times and can actuate taking rotations up to 11,000 revolutions per minute (RPM), in the case of ERMs, and oscillations of the order of few hundreds of Hertz.

**Limitations:** Vibration comes often with undesired noises or sounds. While this is mitigated by rubber-made absorbing structures often integrated in the motors, use cases need to consider this aspect. While vibrotactile actuation offers the opportunity to explore a particular type of haptic feedback, the use of small motors limits the generated effects, in terms of amplitude, duration, and intensity perceived. To create vibration sequences, several motors are needed, provided integration software and hardware development efforts are carried out. The actuators often require extra drivers to widen the operating regime possibilities while maintaining electrical safety standards. As generally advised in the case of feedback modalities applied to the body, haptic feedback actuation has to go hand in hand with user experience studies, since prolonged exposure and certain placements can lead to discomfort.

### 4.4. Temperature Actuation

**How it works:** Temperature actuation uses heating and cooling elements to stimulate communication using heat passed by haptics, that is, through our sense of touch. When used with biosensors, it can use the properties of the heating/cooling dynamics of the material to convey characteristics of the biosignal being tracked.

**What:** Temperature actuation is a type of communication that is used as feedback drawing upon the human haptic (touch) sense. The properties of the temperature feedback depend on the materials that are used to convey the features of the information (e.g., biosignals, behavioral data, etc.) of interest. Most commonly used approaches rely on the conversion of an electrical current input into heat/cold outputs, mainly by using resistive elements that heat up when current flows through. Examples of these are nichrome wires, conductive threads, conductive fabrics, and thermoelectric coolers (Peltier elements).

**Where:** The nature of the heating elements determines where the temperature actuation can be placed. The flexibility of wires and fabric has led to many developments that extend wearable capabilities, producing smart garments that lie close to the skin. Implementation possibilities of these technologies comprise patches and configurations to be mounted in accessories (bags, caps, etc.), among others. In applying heat or cold, placement plays a key role given the different perceptual and comfort ranges that exist throughout the body skin.

**When:** Typically, heat is an actuation modality that acts slowly. Whereas thermoelectric coolers and nichrome could seem to behave otherwise, being able to be turned on in a fast manner thanks to conduction, there is usually an element that plays a dissipating (slow) role in heat dynamics either using convection or radiation. Heat transfer, hence, usually involves relatively slow dynamics.

**Limitations:** As it is the case with actuation having an effect upon the body, heat/cool feedback has to closely consider the user comfort and perceptual thresholds. Materials’ properties (mainly heat conductivity) constrain the possibilities in terms of time. While options like actuation upon wide-areas or multi-actuator sequences emerge as interesting actuation paradigms, power requirements remain a challenge. Moreover, sensitivity to temperature varies widely from user to user and is affected by ambient temperature conditions.

### 4.5. Shape-Changing Actuation

**How it works:** Shape-changing actuation uses interfaces that exhibit changes in size, shape, or texture in order to, when linked to feedback, exploit the human visual and tactile perception to convey meanings and information content. By using shape changes that unfold over time, the actuation dynamics are brought forth letting the user be able to play with concepts such as time (increasingly/decreasingly fast, slow, abrupt) and volume or size to depict the desired information.

**What:** Shape-changing actuation interfaces use the change of physical form that, when linked to feedback, provide a certain output conveying meanings and properties of the signal that they are bound to. Shape-changing exploits a combination of the senses of sight and touch to convey meanings intrinsic in the dynamics of the information that they are linked to, such as rapid changes, stability, increase, decrease, and steady growth. Such interfaces, despite the parallelisms found in visual screen-based explorations done in visual computing, are emerging as an alternative, physical, and tangible way of interacting with technological devices [66]. Three of the most widely used examples of shape-changing actuation elements are:

- Shape-memory wire (“muscle wire”, nitinol, flexinol): a unique type of wire, which can be deformed, stretched and bent at room temperature, able to restore its shape when heated (i.e., when exposed to the electrical current). The wire activates rapidly when the electrical current is applied or the wire is heated.
- Linear actuator: a mechanical device that converts electrical current into a linear movement along a given axis, as opposed to the circular motion of a conventional electric motor. When equipped with extra sensors (such as Hall effect sensors) they are able to provide precise information on their length(the absolute position of the moving element on the axis).
- Inflatable shapes: enclosed structures which can be inflated with fluids (typically gas: air, oxygen, nitrogen, helium, etc.), usually accompanied by pressure or volume control mechanisms.

**Where:** In order for the technology to best benefit from the focus on visual and tactile perception, shape-changing actuation implementations must remain under reach (sight or touch) to convey the meanings embedded in the changes of shape. This can entail direct contact with the body, with the potential to increase the felt shape meaning when in contact with a large body area, or where touch sensations are more developed, or within the field of vision of the user.

**When:** The wide range of shape-changing actuation possibilities comes with different actuation timings in it. While it is possible to work with shape-memory wires that are rapidly heated or compressed gas or pumps that quickly fill up a given inflatable, the time affordances of this kind of actuation do not generalize. Linear actuators, for instance, usually require a system of pistons and damping mechanisms that have an impact on the dynamics of the actuation while it unfolds over time. Moreover, it is often the case that the behavior of shape-changing interfaces is not symmetrical, for example, although an inflatable can be rapidly fed air by a pump or a reservoir, deflation valves have their own rules.

**Limitations:** Memory wires, although visually appealing, imply the utilization of high temperatures which challenges the use of haptic shape-changing feedback based on them. In turn, the strains achieved (or pulling forces) are generally weak, often leading to implementations that use several wires. It is very common that the developments using this kind of actuation include the application of protective heat layers. In the case of linear shape-changing actuators, movement is often accompanied by undesired noise and relatively slow dynamics. The actuators themselves, made of rigid moving elements, impose a certain rigidity to the overall actuation. Moreover, multiple units are often needed to create appealing effects. Shape-changing inflatables often present problems of fluid leaks, as well as different asymmetric behaviors for inflation and deflation. These can be tuned by further developments on valves and compartments but requires significant work. Besides, the type of pump poses specific fluid requirements and usually exhibits noise that interferes with the actuation designed.

## 5. Sensing and Actuation under the Soma Design Approach

Our affective technology research aims to create personal technologies that enable self-reflection [6]. With a focus on the body, design research is used as a way to enter introspectively to emotion self-reflection and potentially disrupt the way we relate to our mental well-being with technology-mediated interactions. Technology-mediated interactions, drawing upon ubiquitous computing capabilities (biosensing, wearables, monitoring applications, embodied actuation) could add novelty and be taken further to psychotherapy contexts. In this section, we introduce the design approach taken to accomplish meaningful biosensing-actuation couplings. This comprises the first-person design stance, the somaesthetic design (“design through the body”) approach, and the path to orchestration (i.e., the mechanisms for the coordination and event recognition in technology-mediated body interactions and the connections and sequencing for evocative sensing-actuation experience design).

### 5.1. Designing from a First-Person Perspective

Often in user-centered design, designers conceive, test, and set requirements for the ultimate users that are placed at the center of the design efforts. In doing so (using a third-person perspective), users are relegated to a second line, in which from time to time, designers probe, test and interview the target users iteratively in order to modify and render the design outcome meaningful according to their needs. This approach, however, misses out the potential of stepping into the user’s shoes. The first-person perspective [67], instead, constitutes a way to highlight the designers’ user experiences, paying honest tribute to the potential end-users, and actively engaging in experiencing the design meanings and effects. The designer who follows the first-person perspective embarks in an iterative process of trying, testing, feeling, and evaluating the designed object or interaction. The design is tried by the designer herself/himself. This process provides meaningful insight into what the eventual user could get from the resulting design. When taking the first-person perspective, the designer is seen as the user, since eventually, anyone interacting with the technology shapes its meaning and how the technology behaves.

### 5.2. Somaesthetic Design

Somaesthetic design underscores the need to place importance on the aesthetic aspect of the felt bodily experiences, as a fundamental element of the design process. This is, for us, a great first step to attempt to create embodied technologies or interactions. The chosen approach, consequently, confers in our case a key role to the body in the design of personal technologies for affective health. Somaesthetics, introduced by philosopher R. Shusterman [10] is the result of the efforts of combining the body with aesthetics, with a strong emphasis on how the body plays a major role in how we feel, perceive and think the world. With somaesthetic design, an attempt is made to leverage the role of the feeling body when engaging in design experiences. This strategy, requiring certain training to grasp one’s sensations, control the movement and perceive what we feel, offers a fresh approach to using the body as the main instrument to feel, assess, and appreciate the design affordances. The soma design manifesto [8] highlights, among other aspects, the need to engage slowly in the aesthetic appreciation of the technologies being designed, disrupting the habitual and inspiring users’ drive to obtain interactions—biosignal-mediated in our case—that lead to novel ways to embrace technologies in our lives. Body practices to support the process of getting attuned to one’s sensations are usually employed, as exemplified in Reference [9]. These often further support the notion of estrangement (or disrupting the habitual), that is, how designers can engage in actions, movements, or performances far from the habitual way of carrying them out. By doing so, the intricacies of a certain interaction become exposed, helping its analysis or reaching to novel possibilities to carry it out.

### 5.3. First-Person Biosensing

Having successfully been applied to design workshops that address the effects of actuation-based interaction for embodiment and self-reflection, the somaesthetic design offers a unique opportunity to address the challenging goal of bringing together biosensing and actuation in an evocative way, relevant to the user who would utilize personal technologies for emotional awareness and regulation. In taking a somaesthetic design approach, affective technology researchers find a path to make sensing workable, paving the way for discoveries that support the creation of new relevant technology-mediated experiences as targeted by the AffecTech project [6]. To explore how our internal physiological mechanisms can be revealed via a set of biosignals, a routine for experiencing sensing from a first-person perspective was followed, significantly inspired by previous haptic actuation explorations [48,68] and used in part in Reference [49]. Using the knowledge in biosignals acquisition and relevant information processing the designed routine aims to support the learning of newcomer students to the field of biosignals, potentially adding tangible interaction features that make the topic more accessible. This process was originally thought as having an expert guide on biosignals and a learner that would follow and report on the felt biosensing experiences during a 1-3 hour session depending on the number of different biosignals covered, but in practice moved to a more open exploration scheme based on switching roles where no expert/novice knowledge hierarchy is sought [49]. The first-person sensing experience goes beyond a theoretical explanation and the lab experience with pre-recorded signals or a recording session of biosignals. With this different approach, the students or researchers that want to be initiated follow an introspection process to discover in a deeper way how and what can change the internal mechanisms of the signals and the body. This process is not thought to take place in a formal lab experience or lecture teaching environment, but accompanied by a person experienced both in soma design and biosensing that would revisit the experience and rely on body practices to awaken the somaesthetic appreciation needed for the exercise (see for instance the work of Windlin et al. and Tsaknaki et al. [9,48]).

### 5.4. Orchestration

Orchestration mechanisms try to answer the overarching goal of achieving systems that facilitate the exploration of biosensor data and meaningful representation through actuation that addresses many more modalities than just visual feedback (see Section 4.1). To achieve this facilitation it is crucial to be able to combine and nicely coordinate the relationship between input (biosensors) and output (actuators). In this paper, the term orchestration defines the process of:

- Creating couplings, that is, combining biosensors and actuators in place
- Coordinating the technology-mediated body interactions
- Working on the sequence in which different modalities are addressed via the sensors and actuators. Deciding which one goes first
- Guiding users in understanding the captured biodata through their felt experience by means of the addressed modalities
- Providing the design exploration ground, showing capabilities, limitations and roles of the involved technologies that participate in these interactions
- Potentially laying out machine learning, feature extraction, smart event recognition, or signal processing tools that can be applied to render the interactions more intuitive or meaningful

### 5.5. A Soma Design Example: The Breathing Light, or How Light Actuation Inspires Design

Interaction design research work by Ståhl et al. such as the Breathing Light [69], preceding the cross-disciplinary efforts presented in this paper, proved inspirational to our research. The goal of the Breathing Light prototype is to help the users to find a safe place where they can take a break from daily routines, focus on inner body processes, and reflect. The prototype is built from fabric and string curtains creating a secluded space for the user’s upper body. The Breathing Light system switches the users’ attention to breathing, focusing on the experience of inhale/exhale cycles. Light has been chosen as a modality given its ability to subtly guide the attention of the participants inwards. Technology-wise, the Breathing Light is a lamp with a proximity sensor. The sensor measures the distance between the chest of the user and the lamp, which in practice becomes a breathing sensor under these conditions. The ambient light in the prototype is dimming in accordance with the breathing patterns: exhale with a dim-out and recovering when inhaling. The intensity of the ambient light is high enough to make it possible to follow the light pattern even with the eyes closed but is not high enough to distract the user. The participants reported that when they were lying under the Breathing Light module, they felt enclosed and taken care of. Limitations arose as it was a demanding task to set the timing, intensity, and warmth of the light. However, in turn, this interaction facilitated an intimate correspondence between the perception of the breathing and the light, which meant that the light was perceived as an extension of the body, providing a much richer experience of breathing.

## 6. Results: Designing Biosensing-Actuation Couplings

In the work of *Somadata* [49], soma design sessions and first-person accounts of the user/designer participants are highlighted to understand what constitutes a tangible or “felt encounter” with an otherwise disembodied design material, that is, biosignals. Our design approach is not that of a “solutionist” method that tries to quantitatively acquire data and formally evaluate how a coupling solves a given problem. This avoidance of a solution is a resource that has been leveraged in design fiction [70]. Our research does not try to tackle the recognition of certain given patterns in biodata to, for example, make users optimize behaviors (walking, running or fitness related activities) nor prevent anomalies (heart malfunction, fall detection, stress recognition). We use design topics, or challenges, at most—such as exploring synchrony between peers. We do not work with a given problem. Rather, a qualitative, explorative stance grounded on the body is at the basis of design discoveries that help us look at biosignals as design material to be shaped, changed and integrated in interaction design toolkits instead of taken as a given, unchallenged and immutable. Where we have failed with other coupling attempts, a selection of carefully crafted sensing-output combinations succeed in achieving what we call *soma data*, that is, biodata that is somatically experienced, leading to novel insight, collectively shareable and in line with a design context or goal. Soma data examples in Reference [49] include a mechanism for groups of two people to connect non-verbally through audio and synchronous movements, a way to share muscle activity insight (on the calf muscles) relevant to an activity of crossing a balancing pole and new way to understand EDA data thanks to a haptic heating effect—that we address in more detail. In this paper, we want to open the design space. Hence, we bring to discussion the underlying interaction mechanisms of this kind of experiences. Although prototypes could be evolved into final products and studied quantitatively, the research presented in this paper is, instead, driven by the inspiration gathered from works that use technology to connect experientially to our felt bodies [9,48,68]. We aim to incorporate biosensing in soma design toolkits, as a design material, and discern what is needed to support this design. When used in soma design workshops, first-person somaesthetic accounts of users exploring the couplings are taken to assess whether an input-output connection is meaningful with regard to body self-awareness. Orchestration decisions are integrated in the organization of wired, programmed effects and input-output mechanisms found in the couplings that successfully led us to what we consider interaction design discoveries [49].

**Figure 2:**
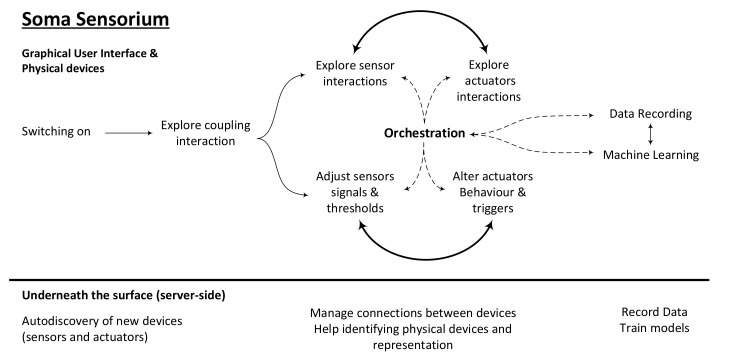
Mechanisms needed for the orchestration of couplings.

However, our interaction design research seems to suggest that better orchestration mechanisms would render our technology-mediated interactions more aesthetic [9,48]. In this line of thought, we have conceptualized a workflow that integrates sensor devices and actuators into a shared network that is controlled through a server, responsible for data transmission between components. In this scenario, the designer has the ability to explore available devices either via a graphical user interface (GUI) or tangible user interface. Furthermore, the available devices can be connected with one another through the GUI to couple input signal streams (such as biosignals) to output signals (like the intensity of a haptic actuator) and allow one to create and fine-tune somatic associations. These associations are designed through defining triggers and responses to signals or even via training machine learning models that react to input signals and act on the output signals. The system should allow for a flexible use of devices, their signals and behaviors to uncover novel interactions (Figure 2). An early example of an interactive visual programming metaphor can be seen in Figure 6.

In this section, we describe *Scarfy*, one of our research outcome biosensing-actuation coupling prototypes (EDA to temperature), as well as current work in Breathing Synchrony and EMG couplings with audio. In our view, a potential orchestration platform should let the users choose what elements are present in every experience, such as sensing-actuation modalities (haptic, sound, light, heat, cold, airflow, shape-changing), count on visual programming interfaces (as used in the EMG-audio feedback experience), enable the possibility to run signal processing code snippets (e.g., breathing synchrony assessment and audio feedback), allow interactions that work more implicitly (movement monitoring, wireless/wearable devices), and set the time sequence structure and order in place, hence shifting the design focus away from the technology constraints and highlighting how experiences are enacted and elements are part of a whole.

### 6.1. Scarfy: A Temperature Scarf to Make Electrodermal Activity Perceptible

**Figure 3:**
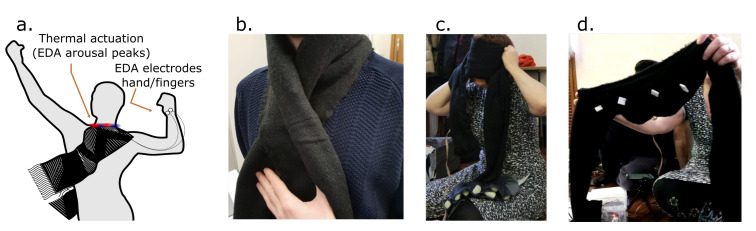
Scarfy: (**a**) EDA heat/cool temperature scarf coupling, (**b**) participants exploring actuation on the neck, (**c**) forehead and (**d**) showing the heating elements.

Inspired by existing research and commercial work on haptic material actuators on the body [43,71], we started exploring different materials and actuators to communicate biodata. We explored materials that are low-cost and safe to use for near body applications that take wearability and comfort into account. After trying out and working with different materials and actuators, that is, thermochromic, heat resistive materials, vibrotactile motors, shape memory alloys [43] and sensors, that is, electrodermal activity, heart rate and breathing, we started preparing a temperature-actuated scarf to promote interpersonal synchrony by linking skin conductance data. This coupling was aimed toward a soma-design session that explored the concept of interpersonal synchrony [49]. We chose the EDA signal, which has been often used to communicate increase and decrease of physiological arousal, to actuate heating and cooling. We used four 20x20 mm Peltier modules in series with a distance of 2.5 cm and enclosed them in a scarf. The resulting artifact can be easily worn on the neck and taken off as shown below (Figure 3).

**Figure 4:**
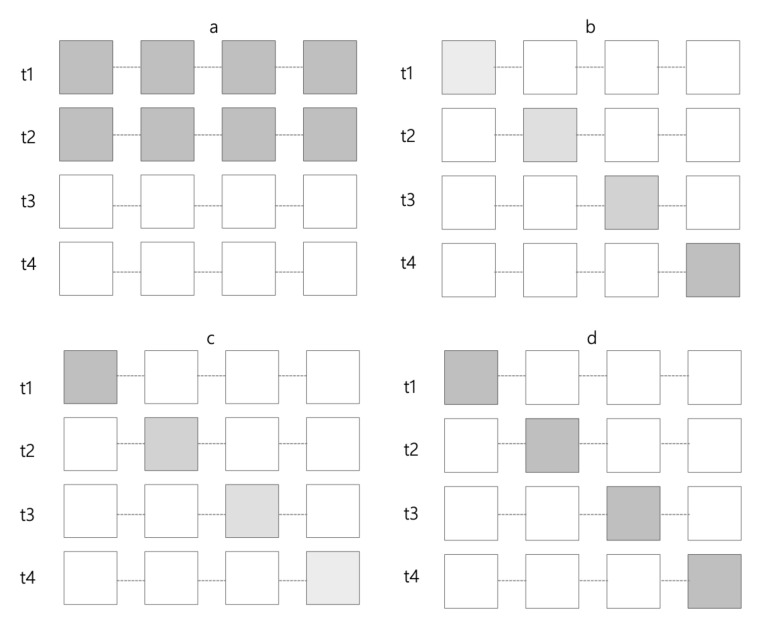
Scarfy EDA-temperature patterns with four Peltier module elements and how they change over time $t_{i}$: (**a**) Appearing/disappearing heat/cool, (**b**) Increasing heat/cool, (**c**) Decreasing heat/cool and (**d**) Moving heat/cool.

The Peltier modules are driven by Arduino boards with motor drivers. Their actuation is triggered by an EDA sensor. To mark the increase and decrease of changes in physiological arousal using temperature, we created four different patterns of heating and cooling as shown (see Figure 4). These patterns are *Appearing/disappearing heat/cool* actuating heat or cold in all the modules at once and then turning them off simultaneously. The second and third patterns as shown in Figure 4b,c are *Increasing heat/cool* meaning that heat or cool slightly turns up module by module, and *Decreasing heat/cool*, that is, gently reducing the thermal effect one by one. The fourth pattern is the *Moving heat/cool* pattern (Figure 4d ) in which thermal actuation is alternated on the modules one by one following a spatial direction and keeping the temperature constant.

The purpose of this coupling and heating and cooling patterns was to communicate increasing and decreasing arousal in interpersonal settings. A set of designers’ first-person accounts and insight on using the prototype in a design workshop focused on synchrony are found in Reference [49]. We wanted to explore how Scarfy can mediate synchrony between people and probe what heating and cooling patterns, intensity and duration would best support this quality. Besides we also wanted to explore feedback around technology *black-boxing* in Scarfy—that is, what design elements should be overtly exposed for customization—how it can be improved, and how it should be used in everyday life settings. Participants in design workshops approached Scarfy by wearing it around the neck, trying out different patterns and positions to feel the increase and decrease of heat and cold. While exploring different patterns and placement we felt that, although subjective, heat and cold have different scales, that is, the sensation of both heating and cooling feels different depending on the parts of the body it is applied to. While exploring the patterns on the body, we discussed how cold feels more pleasant than heat because of the placement of the modules inside a thick scarf fabric which is itself warm. Placing the scarf around the neck, we found that Peltier modules often do not touch the skin and need to be pressed in order to be felt. We were not limited to the neck area only in our exploration. We also explored several other areas such as the forehead (see Figure 3c), back, shoulder, and wrist. While exploring these other placements, we found that the considerable size of the scarf is harder to manage around these other areas. Therefore, we discussed that it would be better to place Peltier modules in smaller strap-on patches that can be placed and taken off easily. It would give us enough freedom to quickly explore patterns on different parts of the body. Finally, talking about arousal and the overall purpose, we discussed that the exploration should shape what meaning we assign to it, that is, whether you are trying to learn about yourself or you are trying to calm yourself down. In fact, ambiguity and the interpretability of electrodermal data have been a recent matter of study in human-computer interaction research [40], with some works questioning the user’s meaning-making processes and challenges when new representations are appropriated, taken outside the lab [72,73]. For Scarfy, any researcher can explore several patterns and needs to figure out which one fits for his/her personal experience, that is, bodily awareness, calming yourself down, feeling your peers’ arousal. Moreover, the interaction described in Reference [49], invites us to rethink how the aspects or features of the signal translated to actuation changes constrict the way biodata is perceived.

### 6.2. Breathing in Synchrony: From Physiological Synchrony to Audio Feedback

**Figure 5:**
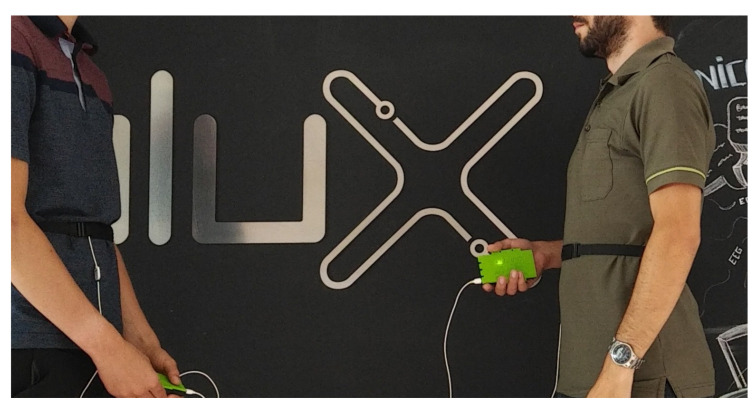
Breathing synchrony-audio experiment, based on the analysis of two BITalino piezoelectric abdominal respiration signals (image showing the two BITalino streaming simultaneously).

This coupling example, drawing upon the psychology concept of therapeutic alliance [74], takes respiration data from two users in the same physical space, where two BITalino devices stream data wirelessly to a host computer. In this example, two users participate in a timed breathing exercise together whilst their individual respiratory patterns are being measured with piezoelectric (PZT) bands placed around the diaphragm (see Figure 5). The data is aggregated on the host computer, executing a script that measures the collective breathing activity. From here, we apply shared biofeedback in the form of sound to stimulate synchrony awareness and physiological dialogue between users over time.

The exploration followed a stage of preliminary research on physiological synchrony features both in published research and drawing upon statistical measurements, potentially generalizable to signals other than breathing. We implemented the computation of linear regression coefficients, cosine similarity and correlations between filtered signal and derivatives. The process for mapping the user’s activity audio output can be split into two main components. First, the device data is transmitted to a Python program, which is used to perform statistical analysis on the incoming signals, calculating a “magnitude of synchrony” using the features listed above. After a fifteen second warm-up period, the system accumulates a sufficient amount of data to determine mutual behavior, and the resulting values are encoded into Open Sound Control (OSC) messages that are continuously streamed to a local address, enabling the designer to map the data to appropriate parameters for sound feedback. With this generic protocol in place, we aim to embrace modularity, and advocate for the experimentation of sonic associations. In our tests, we used Cecilia’s [75] built-in granular synthesis engine; this manipulates the playback of a pre-recorded soundscape divided into independent samples of 10 to 50 milliseconds [76].

### 6.3. Orchestrating an EMG-Audio Feedback Coupling

**Figure 6:**
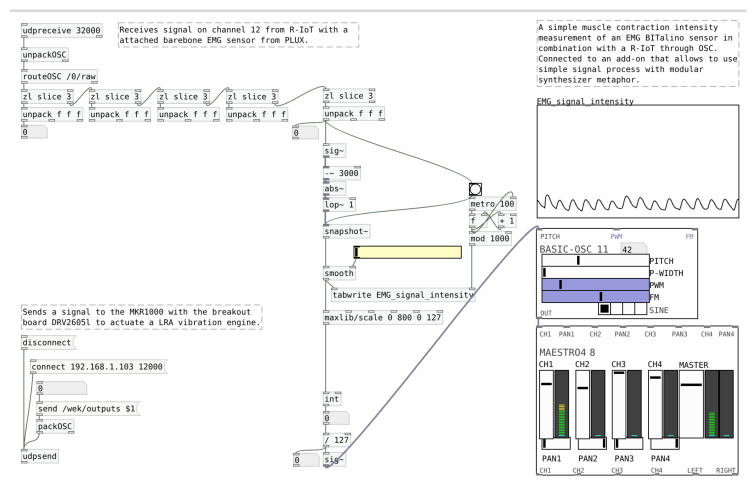
Orchestrating an EMG-audio feedback coupling in a PureData interface patch.

A depiction of an orchestration platform we achieved to create is that of the EMG-audio feedback coupling. Through a visual programming interface called PureData (Pd) [77,78], we connected a muscle activity signal with processing capabilities and a given audio pitch that changes properties according to the biosignal dynamics as muscles are contracted. The interface, shown in Figure 6, presents intuitive elements such as sliders and value boxes that facilitate the decision and modification of the coupling properties. This patch receives the EMG signal from a BITalino R-IoT device, a WiFi-enabled sensor platform, via Open Sound Control (OSC) [79,80] data packets. We mapped the EMG signal to sound in three steps: first, we took the absolute value of the signal—a full-wave rectification; second, we smoothed it with a low-pass filter to remove some of the oscillations; finally, the smoothed signal was mapped to the pitch of a sine wave generator. The higher the measured muscle contraction the higher the pitch the generator produces, and vice versa.

### 6.4. Understanding the Different Input/Output Tradeoffs

Through soma design sessions, we used first-person accounts of the designers or users of the created technology couplings to better understand the drawbacks and benefits that the input and output modalities pose. This subsection lists resulting remarks on the couplings and modalities studied (see Table 2). Scarfy, for example (Section 6.1), draws on previous studies [42,43] that propose novel EDA feedback. Although works such as Reference [41] are interesting in how they look differently at biodata to engage with, in our case we avoid visual feedback and design with the body to investigate effects that can be worn and felt. However, our results, letting us envision the platforms to support the design of couplings, lack the perspective of longitudinal studies highlighting users’ data interpretation [40].

Our claim here is not that a soma design approach is the *best* or most efficient way to design with biosignals. Instead, we argue that soma design provides an interesting way to bridge between engineering- and interaction design perspectives and that this bridge in turn renders novel, creative, and relevant design concepts. In our work, it led to the creation of digitally-enabled experiences that succeeded in making us aware of sensations and reactions of our own bodies as well as those of our peers, at the same time as these explorations pinpointed technological challenges when sensors and actuators were used in ways they were not intended for. It helped us to move away from the predominant health optimization or fitness performance paradigm often present in physical and activity tracking devices that most biosensors are built for. In this sense, it provided a richer space for what biosensing might be used for.

## 7. Discussion

The coupling prototypes presented in this paper, in line with the reflections presented by Reference [49], led to what are arguably design discoveries in combining biosensing with body actuation. These are used to highlight the role of the body and ultimately make us, the users/designers, connect more intimately with it. Soma design is not a shortcut to circumvent the difficulties present when designing with biosensing, but a way to approach them differently. Interaction designers must face the same challenges that engineers or developers struggle with when evaluating what form factor, sampling rate or placement for a sensor is best for a given input or action of interest. Within our soma design exploration, though, issues such as noise in muscle tracking, electrode misplacement, or sensor undersampling that leads to no data variations are experienced through, for example, distorted sounds, excessive vibrations, or changeless temperature feedback, echoing what Fdili Alaoui writes about artists avoiding a problem-solving approach and turning technology resistance into creativity [81]. We believe that instances of couplings that have succeeded in bringing design insight should be integrated into a design or prototyping toolkit. Furthermore, our design approach offers the foundations to successfully integrate different sensing and actuation modalities in a way that is evocative to the body. With regard to Scarfy (Section 6.1), there is a direct link to the works that inspired an EDA coupling [42,43] to be more closely felt on the body emerge from an affective awareness goal. That is also the case of breathing, where Miri et al. [46] show haptic actuation examples with a clear affect intervention workflow. Our approach, instead, is that of supporting the design process. We do so by exploring what effects are possible and using the designer’s own felt body to assess them. The change of focus is relevant. For instance, instead of using feedback as a breathing pacer [ibid.] or affect control mechanism, we delve into the experiential properties of the biosignal at hand and how they are shared or understood collectively. Although there is room for development, the paradigms that we used depict avenues in which we aim to widen the palette of interactions, refine orchestration mechanisms and connect to the underlying ethics of our way of designing bodily awareness or affective technologies.

### 7.1. Orchestration: The Soma Bits Toolkit

**Figure 7:**
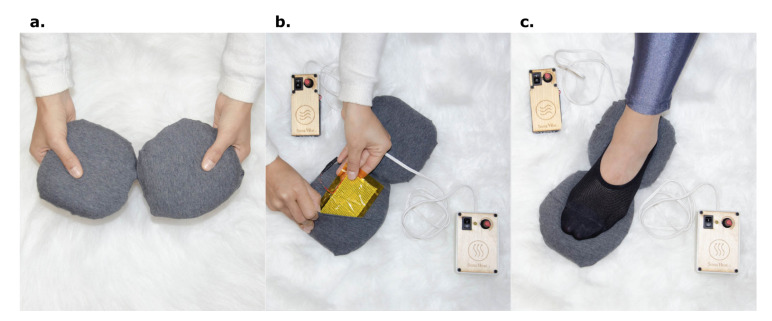
Elements of the Soma Bits design toolkit: (**a**) shapes, (**b**) temperature actuation, (**c**) vibration actuation.

In previous work, we have brought forth the Soma Bits: a prototyping toolkit [48]. Acting as accessible “sociodigital materials”, Soma Bits allow designers to pair digital technologies, with their whole body and senses, as part of an iterative design process. The Soma Bits have a form factor and materiality that allow actuators (heat, vibration, and shape-changing) and sensing (biosensors and pressure sensors) to be placed on and around the body (see Figure 7). They are comprised of a growing library of three-dimensional physical soft shapes, which are made of stretchable textile and memory foam. Each shape has at least one pocket, making it possible to insert different sensing or actuating components. By combining several actuators with shapes, one can orchestrate experiences, and explore the qualities of the sociodigital material directly on the body, by changing the parameters of the sensors and actuators and placing the shapes on different parts of the body.

The Soma Bits are easily (re-)configurable to enable quick and controllable creations of soma experiences which can be both parts of a first-person approach as well as shared with others. In the case of a first-person exploration, someone can, for example, experience and reflect on the properties of heat actuation on their foot and gain a bodily understanding of a heat modality, which can be later integrated into the design of interactive systems. In the case of exploring somaesthetic experiences shared with others, an example can be that one person feels on their spine the breathing patterns and rhythm of another person, translated into a shape-changing pattern that is experienced through the spine soma shape that is part of the Soma toolkit.

We have taken the first steps towards orchestrating collective behaviors of the Soma Bits by combining different Bits and allowing interaction designers to program complex behaviors (e.g., slowly shape-changing materials that heat up when a user presses on them). To achieve that, we aimed at a protocol and an interface for connecting Soma Bits together. In the middle, between sensing and actuation, we provide an “orchestration unit”. This unit acts as a hub for the sensor and actuator network. The orchestration unit is controllable through OSC (Open Sound Control) [79,80]. This protocol enables the usage of common musical interfaces, such as controllers or sequencers, allowing end-users to program the bits without having to write code. We have also started using supervised learning algorithms to quickly help bootstrap interactions. These algorithms allow for mapping noisy sensor data to actuation, which in turn would allow for more complex behaviors to also be programmed into the bits without writing code. We tested the Soma toolkit focusing mainly on combinations of shapes and actuators during three workshops with interaction design researchers and students from several disciplines. Research purposes were explained to participants, who signed informed consent forms. The first was a one-day workshop at the Amsterdam University of Science that took place in October 2018, in which 30 master’s students engaged in a soma design process having the Soma toolkit as the main medium to explore actuation and bodily experiences around the topic of empathy. The second workshop took place in December 2018 at the Mixed Reality Lab, at the University of Nottingham, UK, and was focused on the Soma Bit shapes addressing the topic of balance. First-person accounts of the designers involved in the study and an elaborate analysis of design outcomes of the workshop can be found at References [49,82]. Together, we explored for three days the Soma Bits in several design contexts, including VR applications and leg prosthetics for dancers. During this workshop, we introduced sensing, apart from actuation, through the BITalino prototyping platform [25,55,56]. We also initiated the design of couplings between sensing and actuation, for example by translating movement through acceleration, to sound. The third workshop deploying the Soma Bits was conducted in February 2019 in Milan (see examples in Reference [49]). In this workshop, researchers from several disciplines including psychology, engineering, and interaction design, experienced different prototypes that were brought to the workshop, in combination with the Soma Bits toolkit. The workshop lasted for a day, synchrony was the main topic underlying the prototype demonstrations, as well as the bodily and technological explorations. As a general reflection we observed that as soon as the Soma Bits toolkit was introduced to the design process, the workshop participants shifted their attention to experiencing the sensing-actuation technology through their bodies, rather than just on a conceptual or verbal level. On a broader level, the Soma Bits toolkit addresses the difficulty we experienced in past soma design processes—that of articulating sensations we want to evoke to others, and then maintaining these experiences in memory throughout a design process. Thus, the Soma Bits enable designers to know and experience what a design might feel like and to share that with others. The Soma Bits have become a living, growing library of shapes, sensors, and actuators and we continue using them in our design practices, as well as when engaging others in soma design processes.

### 7.2. Missing Bits: Shape-Changing Actuation

In the creation of a Soma Design toolkit, we aim for a wide range of tunable modalities for the user to explore and create the effects what communicates best for her/his soma. In this regard, we know that our prototyping efforts fall short on making shape-changing actuation available. HCI research has already shown some of the design potential behind linear actuators and inflatable shape-changing mechanisms that inspire us and guide our future research perspectives.

#### 7.2.1. Linear Actuators

**Figure 8:**
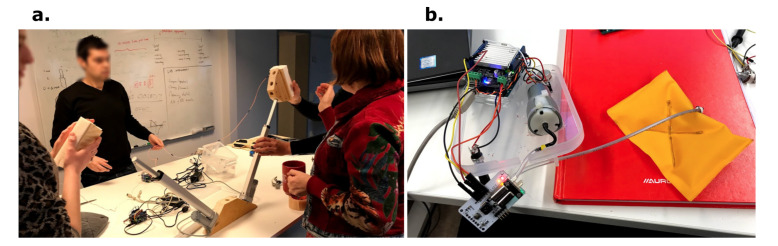
Shape change: (**a**) Prototyping with linear actuators (**b**) Inflatable shape.

The linear actuator serves as a central piece of what we call the Soma Pixel system. Soma Pixel is a modular interchangeable sensor-actuator system (see Figure 8a), inspired by shape-changing projects carried out by MIT researchers from the Tangible Media and the Senseable groups:

- Project Materiable [83,84]
- Project Lift-Bit [85,86]

We aim at having a number of smart modules (“pixels”), capable of sensing the human body (pressure by weight, biosignals) and providing certain actuation. The shape-changing actuator (currently a linear one) serves as a skeleton for the “pixel”. Sensors and other actuators are thought to be located in the upper part of the device. The modules can be easily rearranged in space, while “knowing” their relative position with respect to each other. At the moment, in the Soma Pixel setup, the linear actuator is coupled with a force sensor. Actuation happens when weight/force is being applied to the sensor. This is, nonetheless, accompanied with limitations, that is, the linear actuators cannot move (change length) fast and the motor inside of the linear actuator makes substantial noise when running.

#### 7.2.2. Inflatable Shapes

In an ongoing research line, inflatable shapes are currently being used for constructing a “singing corset” prototype, as well as a part of a newer revision of the Soma Bits toolkit. The corset is built upon an Arduino-powered inflatable that will be integrated with the Soma Bits (see Figure 8b). However, the first iteration of inflatable shapes had difficulties with fast deflation. This was partly solved by adding another air pump, devoted specifically for exhausting air. Experimenting with the addition of separators inside an inflatable shape or splitting it into multiple inflatable sections may further improve exhaust performance. Another limitation is unavoidable minor air leakage, which will happen due to imperfections in manufacturing the actuator (sealing inflatable shape, valve timing).

### 7.3. Extending Orchestration: The Role of Biosignal Processing

A relevant part of our research has focused on the extension of biosignal feature extraction, processing, and analysis in real-time (to enrich real-time feedback possibilities within the lab and in more ecological settings). While initial sensing-actuation orchestration couplings have successfully made use of basic signal processing, further possibilities lie in improving the current algorithms and processing approaches. Any sort of biosignal acquisition, in particular in psychophysiology sensing, can be seen as the process of dealing with sequential and time-series data. Prior to the deployment of feature extraction and selection techniques, data must be preprocessed properly. ECG, EMG, EDA, IMU, respiration, and all the data collected from available sensors are in general sampled at different rates and present different properties that entail signal noise and instances of data that do not conform to (standard) expected representations. These can be seen as artifacts or “meaningless” data. Since the data obtained in real-world ambulatory settings is always noisy, presenting inconsistencies or missing values, preprocessing and cleaning is required (see examples in previous studies we conducted [57]). As it is the case for the features listed in Section 3, time domain and statistical features such as, in the case of designing with breathing and/or heart monitoring (ECG), the mean value of the rates, the mean value of the time between events, the standard deviation of those intervals and the root mean square of successive interval differences are accessible in the wild. Of particular interest are the less apparent frequency-based features which given the demanding spectral analysis computational requirements have just begun to appear in the nowadays more capable out-of-the lab devices. While certain features count on solid research support, pointing at the most scientifically validated ones, attention is given to the exploration of the potential mappings that can be built upon them. The use of multimodal data (namely, the use of several biosensors at the same time), extends the monitoring capabilities that can be brought to the couplings’ design by widening the perspective and being able to keep track of body signals that are not the main focus of the interaction. This allows the designer to put in place validation mechanisms, gaining accuracy, and supporting the claims made with respect to the the targeted biosignal. If orchestration aims at the creation of meaningful technology-mediated interactions, the made connections need to rely on processing capabilities, event detection, and high-level feature extraction to overcome the limitations of too basic couplings that only put in place simple (signal) amplitude-to-intensity mappings. Moreover, the group somaesthetic design appreciation sessions where the felt experiences are brought together and debated (usually relying on inspirational body-centered exercises, reenactions, body sketching tools, and verbal communication) could be significantly enriched with the monitoring of signals acquired during the design experience. As design explorations have already started to show, there seems to be room for the creation of machine learning and processing capabilities that could render interactions with the technology more implicit (also embodied or intuitive).

### 7.4. Extending Orchestration: Machine Learning Capabilities and Personalization

Standard machine learning workflows are usually multi-step and involve the definition of problem-specific feature extraction methods, as well as in-depth expert knowledge of the problem at hand. Deep Learning (DL) [87] techniques, also part of our signal processing research efforts, shift the ML practitioner focus from that of feature extraction methods to feature learning. In particular, in end-to-end learning settings, DL models are directly fed with the raw input signal (i.e., without any form of pre-processing, or at best very mild), and use this to automatically extract (deep) features from it and make predictions based on them [23,88]. Its benefits apply as well to machine learning approaches aimed at improving pattern recognition capabilities of relevance in physiology or affective data [89]. In cases in which the input signal is high-dimensional or when timestamps are a relevant characteristic of the problem at hand (e.g., biosignals, multimodal), DL methodologies have been shown to outperform traditional pattern recognition techniques. While research for real-life DL deployments is still under progress, with algorithm computational resources being one of the main limitations nowadays, DL approaches emerge as mechanisms to overcome the difficulty of coming up with sensible hand-crafted features for a certain classification problem at hand. Provided advances towards DL in ambulatory scenarios are made, orchestration design (of sensing-to-actuation couplings) can potentially benefit from DL feature learning by lowering the technology expertise burden on the designer/experimenter side. Overall, this would allow experience design to focus more specifically on the affordances of the interaction rather than the processing mechanisms. To this end, work on Convolutional and Recurrent Neural Networks (CRNN) has shown to be able to deploy a Neural Network paradigm that obtains classification/recognition performance improvements by algorithm personalization, that is, avoiding specific calibrations to be done according to the user. Typically, personalized Machine Learning models are simply the idea of learning individual behaviors that train a model using the data collected only from the subject. These models are usually customized to the requirements of each individual, imposing requirements to consider the individual differences, yet still using data collected from the population. To some extent, new deep learning paradigms are circumventing these requirements.

In Section 3, we describe how embodied sensor technologies react differently in accordance with the unique biological characteristics of the body. Similarly, the perceived impact of a given actuation mechanism—as those described in Section 4—largely depends on the sensitivity to a given stimulus, as well as the natural bodily variations between different users. With this considered, we recognize the necessity to attune the system’s parameters in order to produce mappings that facilitate meaningful interactions that are not overly obtrusive. While auto-calibration mechanisms have been implemented in the previous examples, which typically define and minimum/maximum parameter ranges, we foresee an extended benefit in adopting Interactive Machine Learning (IML) [90] frameworks as means to foster perspectives respecting body pluralism. Existing frameworks such as *Wekinator* [91] and *Teachable Machine* [92] facilitate the design of classification and regression-based mappings that are initiated and iteratively adjusted with example data provided by the user (e.g., Reference [93]). Furthermore, we would like to explore the use of Interactive Machine Learning to develop novel coupling relationships that go beyond linear mappings, as well as intuitive mappings between multimodal inputs and multi-dimensional outputs.

In our sound-based examples, visual programming environments heavily assisted the orchestration process. In both cases, the systems enabled users to visualize a continuous stream of mappable data in real-time, clearly exposing any unexpected behavior that may occur (for example, with the displacement of sensor electrodes). The node-based functionality of the frameworks allowed for a coherent representation of the dataflow and signal processing steps in order of execution, less abstract compared to a code-based script. During the process of developing the system, a user interface is generated in parallel on-the-fly as each node presents a GUI element that grants the designer access to parameters such as scaling and smoothing coefficients. In Section 6.2, a basic interface allowed users to test and compare a set of algorithms for sound mappings. This workflow can be beneficial for rapid experimentation with a variety of parameters and signal processing techniques that influence the interactive experience. It also presents a convenient solution for fine-tuning a complete system according to the user’s experience.

### 7.5. Ethical Underpinnings

We have provided examples of research on sensing and actuation technologies, first-person somaesthetic approaches and experiments to foster the design and research of self-awareness (embodied, wearable) technologies with the potential to support self-reflection, emotion regulation, and affective health. In any design process, and particularly for technologies that may be used in affective or health contexts, it is important to consider the ethical implications of design, use, and research. Ethics simply concerns what is *good*, with a utilitarian perspective dealing with the greatest good for the greatest number of people. To assist in ethical decisions and practices, ethical frameworks outline key concepts such as beneficence and nonmaleficence, justice, responsibility, autonomy, privacy and confidentiality, and respect for the rights and dignity of others [13,14,94,95,96,97]. By reflecting on these ethical principles and standards, designers and researchers can ensure that their processes, technological developments, and research are done ethically and for the greater good.

The concept of the first-person somaesthetic design reflects qualities of good ethical practice through its focus on experiencing each aspect of the technology to facilitate its appropriate use, integration, and development. This extensive design process explores the benefits and potential adverse effects of the various sensors and actuators and uses this to shape orchestration and future design. Guiding users in interpreting the captured data throughout the felt experience and exposing or creating the meanings attached to personal sensing is a deliberate effort to make interactions intuitive but conspicuous. In Reference [67], designing with the body is seen as a route for designers to harmonize with their felt experiences, an alternative of particular relevance in light of the implicit interaction direction that personal tracking technologies seem to move into. The slow and deliberate soma design methods also reinforce the careful consideration of how technology may impact experience, and therefore the potential effects this may have on future users. Part of our research on orchestration, attempts to provide the design exploration ground that would show capabilities, limitations and roles of the involved technologies that participate in the designed interactions, in line with the first-person perspective. It is important to acknowledge that when stepping into the user’s shoes, designers jump to the firing line. Potential effects for the user are experienced firsthand. Another ethical strength lies in the acknowledgement of the need for variety and customization of these experiences to suit the individual end-users and their specific needs, as avoiding the blindness for body differences is advocated early on in first-person work [67]. This is important for inclusive and diverse technologies which must consider not just individual differences across users, but also users with additional needs or impairments who may be disadvantaged or excluded from technologies which focus on only one modality, such as visual interfaces. The ethical somaesthetic design will involve consideration and incorporation of ethical principles and practices in the design process from conceptualization and throughout the design and development lifespan. Designers and researchers should be familiar with core ethical principles and should reflect on how these may shape their design practices, research, and technological developments. Research examples such as the work of Balaam et al. [35], point at ways to challenge the design practice, especially in emotion work, and avoid engaging participants by default but questioning and justifying their engagement. Autoethnographic and first-person design are seen as promising alternatives. Future research should explore how to incorporate user-centered design within the first-person design process. This will increase the validity of the premise that first-person experiences can be used to create devices to serve the diversity of human experiences. This is especially crucial for devices that may be used for emotion regulation or affective health, where different users may have different experiences, needs, and risks based on their unique histories and circumstances. Experiments such as the design explorations and couplings described in this paper can, therefore, be adapted to involve persons with varied lived experiences to explore how their experience of the soma toolkit or other technologies may differ from those of the designers. This is further connected to concepts of beneficence, non-maleficence, and justice which encompass issues related to benefits, risks, safety, fairness, and equal access for all. Technology offers opportunities to reduce barriers but to do so, design and development must consider how to deliver innovative and impactful technology while still being accessible and affordable. Designers also have a responsibility to consider the intended and unintended potential consequences of any new technology, and the need for appropriate design, guidance, and support to ensure safe use. While discussions of impact, effects, and outcomes are centered on end-results, it is crucial for these to be considered at the beginning of the design process and throughout to ensure the creation of good and safe technology. Also important to consider are issues of data security, privacy, and ownership of personal data, with the safe and secure handling of biosensor data an important design consideration with ethical and legal implications for its use and misuse. Finally, while somaesthetic couplings may offer opportunities for self-awareness and emotion regulation, designers must consider the balance between optimizing and pathologizing typical human experiences, as well as the potential stigma in encouraging the tracking and monitoring of affective health. As with all ethical issues, this must be considered throughout the design process. Like the somesthetic design, the ethical design must be a continuous process integrated throughout all aspects of the design experience and production.

## 8. Conclusions

Research on mobile and wearable technologies that track behavioral, psychological, and contextual signals has recently gained momentum in the field of mental health. At the same time, the rise of personal sensing has garnered the interest of HCI research. In this paper, we approached the design of sensing-actuation experiences intended for rich embodied interactions with relevance to affective health. To achieve this, we adopted first-person soma design to integrate biosignals that are commonly used in ubiquitous low-cost personal sensing together with actuation mechanisms studied in HCI. Our design exploration, giving special attention to the sentient body and acknowledging alternative ways to address affect within interaction, culminated with a set of coupling examples, in which we demonstrate data mapping strategies between various devices in the context of bodily and emotion awareness. The soma design approach applied to the creation of biosensing-actuation couplings for affective and self-awareness experiences is the main contribution of this paper. Through the couplings, we arrived at the concept of orchestration, defining the ways in which body input-output systems and meanings are put in place, the range of mappings and how they unfold. Soma design is a theoretically robust design approach that helps us sketch experiences to develop a (not necessarily dialogue-oriented) toolkit to facilitate creating affective technologies grounded on the body and enhanced by biosignals that are made available as design material. As a design toolkit, the examples created so far are instances of a wider collection of tools. The findings of our design explorations have unveiled a set of research directions (or requirements) to pursue in order to achieve broader orchestration mechanisms:

1. Further work on real-time machine learning tools to train biosignal-based input-output effects on the fly (in line with research in interactive machine learning [90,91,92]) and extend options to customize the features recognized and feedback received, according to the user preferences
2. Continue developing and testing programming interfaces that not only enable setting new sensing-actuation connections but leverage the user configuration and control while a coupling is being explored
3. Carry out more studies on multi-sensor experiences, as these point to the need for more advanced real-time signal processing and the rich multi-user interactions where outputs are the result of user collaborations or shared, bodily understanding of biodata

This insight aims to inspire developments in affective technologies and invites the joint work of engineering, interaction design, or even clinical disciplines that are traditionally disconnected from one another. Moreover, our discussion points at current limitations and paves the way for future research. We indicate sensing-actuation modalities that have been underexplored, then we consider the potential benefits of integrating refined machine learning algorithms and (developing) new orchestration interfaces to assist and democratize the crafting and customization process. As somatic perspectives are becoming more incorporated in areas of interaction design (research) and embraced with rigor, we foresee valuable intersections in other research domains.

## Figures and Tables

**Table 1:** Parameters and type of energy measured through body sensing. Adapted from Reference [50].

Energy	Changing Parameter	Measurement Examples
Mechanical	Position, force, torque, pressure	Muscle contractions, cardiac pressure, muscle movement
Electrical	Voltage, charge, current	EMG, ECG, EEG, EDA, EOG
Thermal	Temperature	Surface body temperature
Chemical	Concentrations, exchanged energy	pH, oxygen, hormonal concentrations

**Table 2:** Technology drawbacks and benefits.

	Drawbacks	Benefits
**EDA temperature**	Limited EDA placement High power supply needs Circuitry-dependent orchestration High temperature safety risks Non-symmetrical effects (increase/decrease, heat dissipation)	EDA data is shown tangibly (not only as peaks building up but also dissipating) Low sampling rate, easily trackable with averages Slow signal in line with the deliberate soma design stance On-the-body effects (perceptible and physically grounding) Easy to adjust, put on and take off in case of discomfort
**Breathing synchrony input**	Advanced processing features that capture synchrony and multi-sensor behavior Piezoelectric breathing sensor limitations (precise breathing rates but inaccurate breath holding detection)	Multi-sensor (allowing multi-user and synchrony studies) Highly controllable (useful for affective tracking but also interaction controls, for example, breathing amplitude and rate)
**EMG input**	Placement for specific muscle tracking (trial and error needed) with high sampling rates (rapid muscle activity is precisely captured and multi-EMG muscle group and articulation monitoring possible)	Low cost sensors with high sampling rates (rapid muscle activity is precisely captured and multi-EMG muscle group and articulation monitoring possible Simple processing (signal energy and envelopes) to detect EMG bursts
**Audio feedback**	Off-the-body actuation (needs a context or activity to relate to the physical body)	Highly developed human hearing (high perception of pitch and rhythm changes) Large consumer electronics audio possibilities (wireless speakers and headphones) Many programming interfaces for audio. Music development area (many programming languages, libraries, platforms) Existence of audio processing libraries in visual programming platforms → address orchestration platform GUIs
